# Interaction between Tumor-Associated Dendritic Cells and Colon Cancer Cells Contributes to Tumor Progression via CXCL1

**DOI:** 10.3390/ijms19082427

**Published:** 2018-08-16

**Authors:** Ya-Ling Hsu, Yi-Jen Chen, Wei-An Chang, Shu-Fang Jian, Hsiao-Li Fan, Jaw-Yuan Wang, Po-Lin Kuo

**Affiliations:** 1 Graduate Institute of Medicine, College of Medicine, Kaohsiung Medical University, Kaohsiung 807, Taiwan; hsuyl326@gmail.com; 2 Graduate Institute of Clinical Medicine, College of Medicine, Kaohsiung Medical University, Kaohsiung 807, Taiwan; chernkmu@gmail.com (Y.-J.C.); 960215kmuh@gmail.com (W.-A.C.); chienfang1216@gmail.com (S.-F.J.); puffmilkian@gmail.com (H.-L.F.); 3 Department of Physical Medicine and Rehabilitation, Kaohsiung Medical University Hospital, Kaohsiung 807, Taiwan; 4 Division of Pulmonary and Critical Care Medicine, Kaohsiung Medical University Hospital, Kaohsiung 807, Taiwan; 5 Division of Colorectal Surgery, Department of Surgery, Kaohsiung Medical University Hospital, Kaohsiung 807, Taiwan; 6 Center for Infectious Disease and Cancer Research, Kaohsiung Medical University, Kaohsiung 807, Taiwan

**Keywords:** colon cancer, dendritic cells, CXCL1, miR-105, Parathyroid hormone-related protein

## Abstract

Crosstalk of a tumor with its microenvironment is a critical factor contributing to cancer development. This study investigates the soluble factors released by tumor-associated dendritic cells (TADCs) responsible for increasing cancer stem cell (CSC) properties, cell mobility, and epithelial-to-mesenchymal transition (EMT). Dendritic cells (DCs) of colon cancer patients were collected for phenotype and CXCL1 expression by flow cytometry and Luminex assays. The transcriptome of CXCL1-treated cancer cells was established by next generation sequencing. Inflammatory chemokine CXCL1, present in large amounts in DCs isolated from colon cancer patients, and SW620-conditioned TADCs, enhance CSC characteristics in cancer, supported by enhanced anchorage-independent growth, CD133 expression and aldehyde dehydrogenase activity. Additionally, CXCL1 increases the metastatic ability of a cancer by enhancing cell migration, matrix metalloproteinase-7 expression and EMT. The enhanced CXCL1 expression in DCs is also noted in mice transplanted with colon cancer cells. Transcriptome analysis of CXCL1-treated SW620 cells indicates that CXCL1 increases potential oncogene expression in colon cancer, including *PTHLH*, *TYRP1*, *FOXO1*, *TCF4* and *ZNF880*. Concurrently, CXCL1 displays a specific microRNA (miR) upregulated by the prototypical colon cancer onco-miR miR-105. Analysis of publicly available data reveals CXCL1-driven oncogenes and miR-105 have a negative prognostic impact on the outcome of colon cancer. This study indicates a new mechanism by which the colon cancer milieu exploits DC plasticity to support cancer progression.

## 1. Introduction

Colorectal cancer (CRC), the third most common cancer with over 1.2 million new cases diagnosed worldwide annually, accounts for 10% of all cancer cases [1,2]. The majority of metastatic CRC patients remain incurable, with a median survival of less than 24 months [3]. Cancer recurrence and metastasis are the most important survival-influencing factors of CRC [2,4]. Unfortunately, up to half of CRC patients will develop recurrent cancer after treatment that was intended to cure their original disease [4]. Continuing research to better understand CRC biology continues to be important in guiding the development of more effective therapies.

Malignant cells are phenotypically and functionally heterogeneous in the tumor microenvironment, which has been shown to be a major contributor to the onset and progression of various malignancies, including colon cancer [5,6]. Dendritic cells (DCs) are characterized as heterogeneous and vastly plastic immune cells with a predominant role in regulating immune responses [7,8]. Accumulating evidence indicates that DCs located in the tumor microenvironment not only impede anticancer immunity, but are also active facilitators in the progression of various cancers [9,10,11]. The majority of tumor-associated DCs (TADCs) in colon cancer have been shown to display an immature state, with a substantial subset expressing high levels of inflammatory cytokines/chemokines or the T-cell inhibition ligand PD-L1 [12,13]. Communication between colon cancer cells and DCs is complex and yet to be fully elucidated. Understanding the interactions between DCs and tumors is important for comprehending the mechanisms of tumor immune surveillance and will provide prospective targets for innovative therapeutic strategies aimed at reprogramming their abnormal phenotypes toward effective anti-cancer activity.

A growing body of studies has shown inflammatory cytokines and chemokines can act as tumor growth and survival factors and promote tumor progression and metastasis by increasing angiogenesis and suppressing immune-mediated tumor elimination [14,15,16]. Elevated chemokine (C-X-C motif) ligand 1 (CXCL1) levels are found in CRC, and these increased levels are positively associated with cancer stage, metastasis and poor survival rates [17,18]. A recent study has revealed that CXCL1 contributes to the formation of a pre-metastatic niche in the liver by recruiting chemokine (C-X-C motif) receptor 2 (CXCR2)-positive myeloid-derived suppressor cells [19]. However, the source of elevated CXCL1 and the underlying mechanisms responsible for colon cancer progression remain elusive. In this study, we investigate whether colon cancer cells alter the DC phenotype toward pro-tumoral activity and how TADC-derived factors promote cancer progression. Our study provides new insight to understanding the unique crosstalk that accounts for cancer promotion by DCs in colon cancer.

## 2. Results

### 2.1. The Change in Dendritic Cell (DC) Phenotypes in Colorectal Cancer Patients

**Figure 1:**
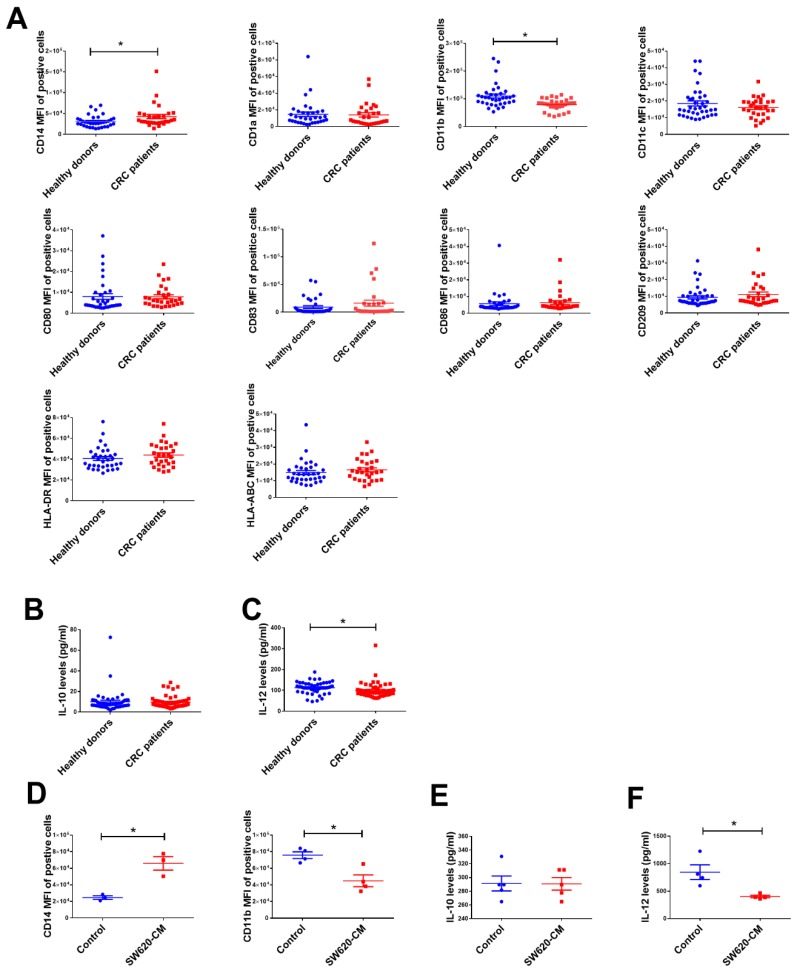
The dendritic cell (DC) phenotype of colorectal cancer (CRC) patients. (**A**) The changes in surface marker expression of DCs isolated from healthy donors and patients with CRC. The levels of IL-10 (**B**) and IL-12 (**C**) in DCs. CD11c^+^ cells were isolated from healthy donors and patients with CRC, the surface markers of the cells were determined by flow cytometry after staining (shown as mean fluorescence intensity (MFI)), and the levels of IL-12 and IL-10 were measured by Luminex assay. (**D**) The phenotype of SW620-conditioned tumor-associated DCs (TADCs). The levels of IL-10 (**E**) and IL-12 (**F**) in TADCs. CD14^+^ monocytes were cultured in RPMI-1640 containing GM-CSF (10 ng/mL) and IL-4 (10 ng/mL) with or without L-15 medium or SW620-condition medium (SW620-CM, 50%) for five days. Results are representative of at least three independent experiments. Each value is the mean ± SD of three determinations. * Significant difference between the two test groups (*p* < 0.05) (*).

Our previous study showed that tumors can cause phenotypical and functional alterations in DCs in cancer [10]. Consequently, we assessed whether colon cancer cells also cause dysfunction of DCs. To do so, we collected CD11c^+^ cells from healthy donors (*n* = 33) and CRC patients (*n* = 30), then assessed various DC-related surface markers and cytokine production. As shown in Figure 1A, significant alteration was noted for the expression pattern of CD14 and CD11b in the DCs of patients with colon cancer. Compared with the DCs of healthy donors, CD11b expression was lower in the DCs of CRC patients, and a subpopulation of these cells still showed significant expression of CD14. In addition, DCs isolated from patients with CRC produced lower levels of anti-tumor cytokine IL-12, although there was no significant difference in the production of immunosuppressive cytokine IL-10 (Figure 1B,C).

To mimic the effects of CRC on the changes in DCs, we derived DCs from CD14^+^ monocytes in the presence of SW620-conditioned medium (CM). As expected, monocytes cultured in the presence of SW620-CM differentiated into CD14^+^CD11b^low^ DCs (defined as TADCs) (Figure 1D). In addition, the expression of IL-12 in the DCs conditioned by SW620-CM (50%) was lower than that of DCs derived from control medium (50% L-15 medium), while there was no significant difference in the levels of IL-10 (Figure 1E,F). These data coincide with human CRC patient data.

### 2.2. SW620 Cells Increased the Expression of CXCL1, 2, and 3 in Tumor-Associated DCs In Vitro and In Vivo

**Figure 2:**
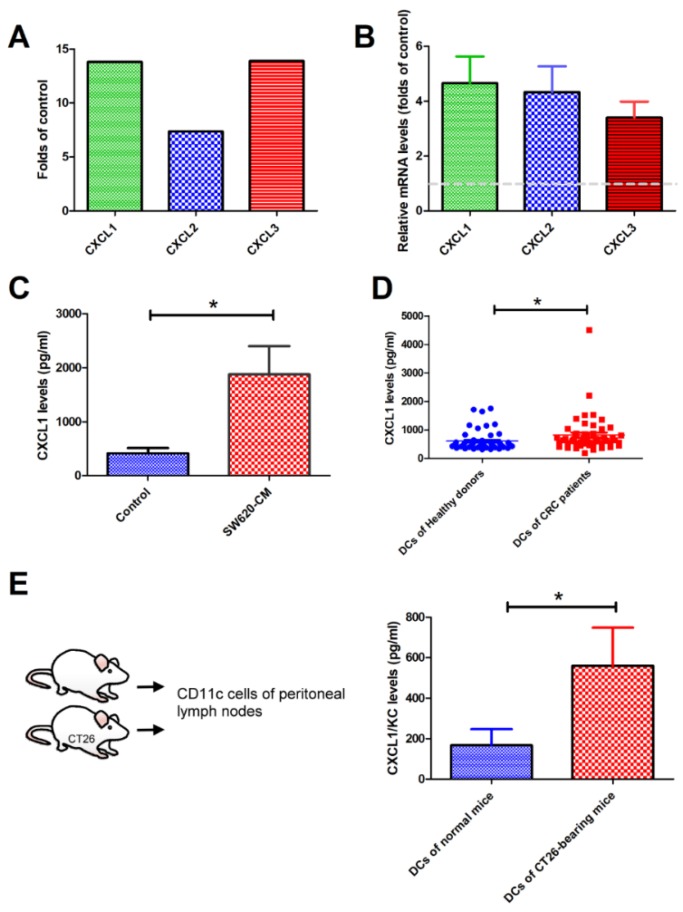
Upregulation of CXCL1 in DCs isolated from mice and patients with CRC. Increased CXCL1, CXCL2 and CXCL3 (**A**) in SW620-conditioned TADCs, as determined by microarray. Elevated CXCL1 in SW620-conditioned TADCs at mRNA (**B**) and protein (**C**) levels. TADCs were generated by culturing CD14^+^ monocytes with RPMI, L-15 medium (50%), and SW620-CM (50%) and presenting in GM-CSF (10 ng/mL) and IL-4 (10 ng/mL) for five days. The expressions of mRNA and protein were assessed by microarray, qRT-PCR and Luminex assays. (**D**) The level of CXCL1 in the DCs isolated from patients with CRC. CD11c^+^ cells were isolated from healthy donors and patients with CRC, and the levels of CXCL1 were measured by Luminex assay. (**E**) The levels of CXCL1 in DCs isolated from colon cancer-bearing mice. Mouse colon cancer CT26 cells were injected into mice via intraperitoneal injection. After 14 days, peritoneal lymph nodes were harvested. CD11c^+^ DCs were isolated from the lymph nodes, and the culture medium collected after 24 h incubation. CXCL1 levels were determined by ELISA. Results are representative of at least three independent experiments. Each value is the mean ± SD of three determinations. * Significant difference between the two test groups (*p* < 0.05) (*).

To investigate whether TADCs contribute to shaping the tumor microenvironment, we assessed the gene profile of SW620-TADCs using microarrays. The data showed that several soluble factors increased in SW620-TADCs [10]. Among these upregulated genes, the levels of CXCL1, CXCL2 and CXCL3 increased 13.8, 7.4 and 13.9-fold in SW620-TADCs, respectively (Figure 2A). qRT-PCR and ELISA analysis revealed that the expression of CXCL1 increased in SW620-TADCs, at both the mRNA and protein levels (Figure 2B,C). Moreover, increased levels of CXCL1 were also found in the DCs isolated from patients with CRC (Figure 2D). We also used animal experiments to determine whether colon cancer increased CXCL1 expression in DCs in vivo. We injected the mouse colon cancer cell line CT26 into mice, then allowed the cells to develop for 14 days. ELISA analysis has also shown that colon tumor-infiltrated CD11c^+^ DCs produce elevated levels of CXCL1 protein, compared to the DCs isolated from normal mice (Figure 2E). These data coincide with in vitro and human CRC patient results.

### 2.3. CXCL1 Increased the Cancer Stem Cell Properties of SW620 Cells

**Figure 3:**
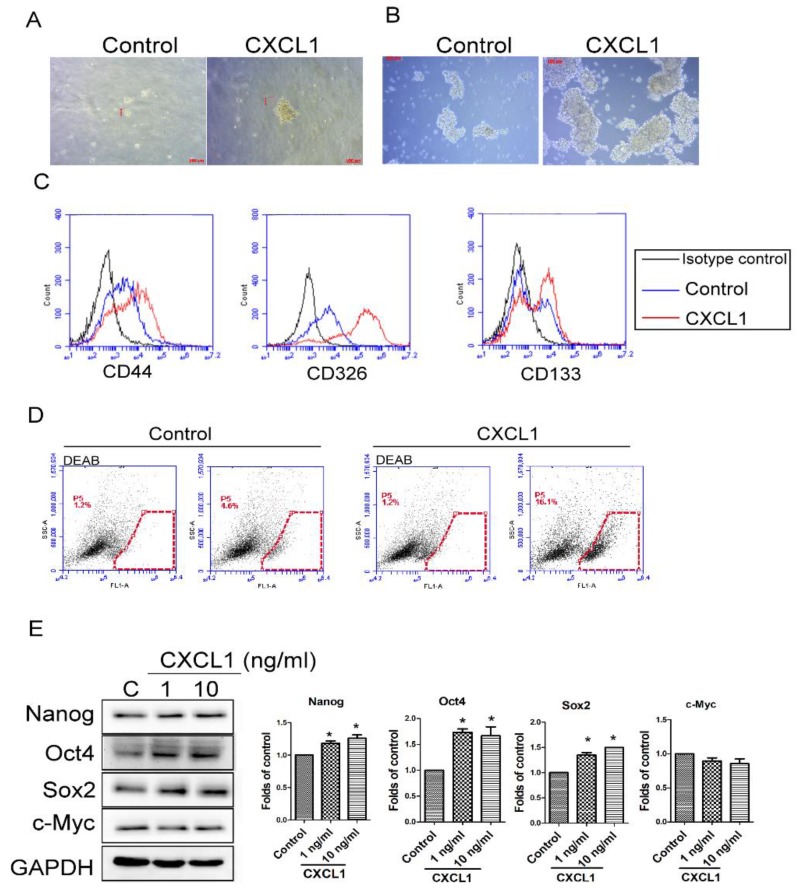
CXCL1 increased cancer stem cell (CSC) properties in SW620 colon cancer cells. Anchorage-independent growth (**A**) and tumor spheroid formation (**B**) of CXCL1-treated SW620 cells. The expression of CD44, CD326 and CD133 (**C**) and aldehyde dehydrogenase (ALDH) activity (**D**) in tumor spheroids. SW620 cells were cultured with CXCL1 protein in soft agar (0.4%) or ultra-low attachment wells for 40 and 10 days, respectively. The tumor spheroids were counted by microscope. ALDH activity and surface markers of tumor spheroids were determined by ALDEFLUOR assay and flow cytometry. (**E**) Level of CSC-related transcription factors in tumor spheroids. Results are representative of at least three independent experiments. Each value is the mean ± SD of three determinations. * Significant difference between the two test groups (*p* < 0.05) (*).

Elevated cancer stem cell (CSC) properties in malignancy correlates positively with cancer progression and metastasis [20]. Therefore, we investigated the influence of TADC-derived CXCL1 on CSC characteristics. Soft agar colony formation analysis revealed that CXCL1 significantly increased the ability of individual SW620 cells to survive and form colonies on soft agar (Figure 3A). Results of tumor spheroid assays reveal that CXCL1 enhanced SW620 cells to form tumor spheres, in contrast with control cells (Figure 3B). The colony and spheres formed from the CXCL1-treated SW620 cells were larger in diameter compared to colony and spheres of control SW620 cells. Corresponding with our results of enhanced anchorage-independent growth, CXCL1 also increased the expression of CD44, CD326 and CD133, well-known cancer stem cell markers in tumor spheroids (Figure 3C). The activity of aldehyde dehydrogenase (ALDH), another critical CSC indicator, was enhanced in tumor spheroids cultured with CXCL1 (Figure 3D). Moreover, the expression of CSC-related transcriptional factors, Nanog, Oct4 and Sox2, were enhanced by CXCL1 in SW620 tumor spheroids (Figure 3E).

### 2.4. CXCL1 Enhanced Cell Migration and Epithelial-to-Mesenchymal Transition in SW620 Cells

**Figure 4:**
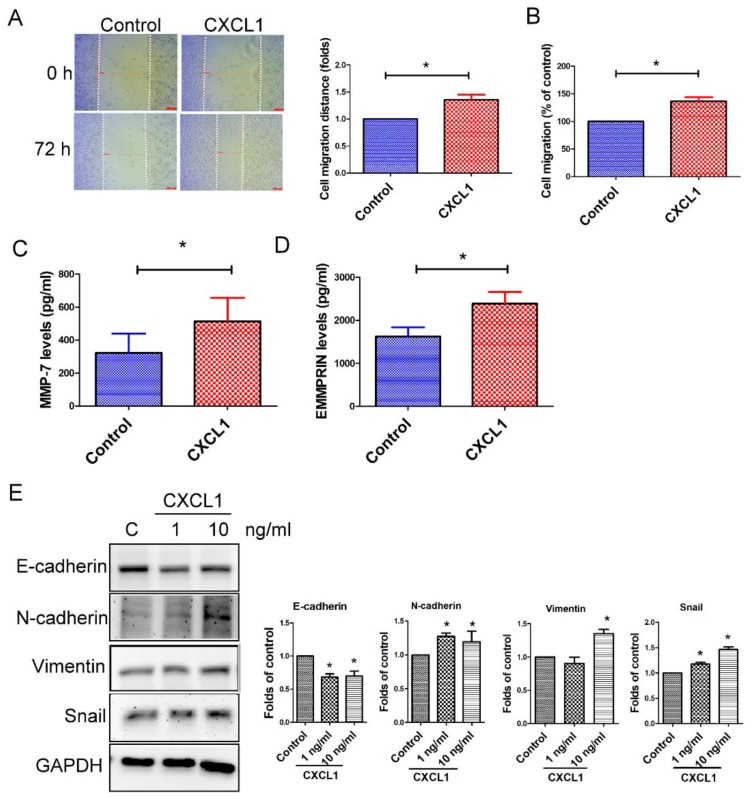
CXCL1 enhanced cancer progression in SW620 cells. CXCL1 increased cell migration, as determined by wound healing (**A**) and transwell (**B**) analysis. The migration ability of SW620 cancer cells was assessed by wound healing assay and transwell assay. CXCL1 acted as a chemoattractant for cancer migration in the transwell system for 24 h. The effect of CXCL1 on the expression of MMP-7 (**C**), EMMPRIN (**D**) and epithelial-to-mesenchymal transition (EMT) (**E**) in SW620 cells. SW620 cells were treated with CXCL1 for 24 h, and the levels of various MMPs and EMT markers were determined by Luminex assay and Western blot. Results are representative of at least three independent experiments. Each value is the mean ± SD of three determinations. * Significant difference between the two test groups (*p* < 0.05) (*).

We next assessed the effect of TADC-derived CXCL1 on colon cancer tumor aggressive behavior. CXCL1 increased cell migration of SW620 cells, as determined both by wound healing and transwell analysis (Figure 4A,B). A Luminex assay reveals that CXCL1 increased the expression of MMP-7 and EMMPRIN, but not other MMPs (MMP-1, -2, -3, -8, -9, -10 and -12, data not shown) in SW620 cells (Figure 4C,D). Furthermore, CXCL1 also caused SW620 cells to undergo epithelial-to-mesenchymal transition (EMT), including the downregulation of epithelial marker E-cadherin, and upregulation of fibroblast markers N-cadherin, vimentin and transcription factor Snail (Figure 4E).

### 2.5. The Transcriptome of CXCL1-Treated Colon Cancer Cells

**Figure 5:**
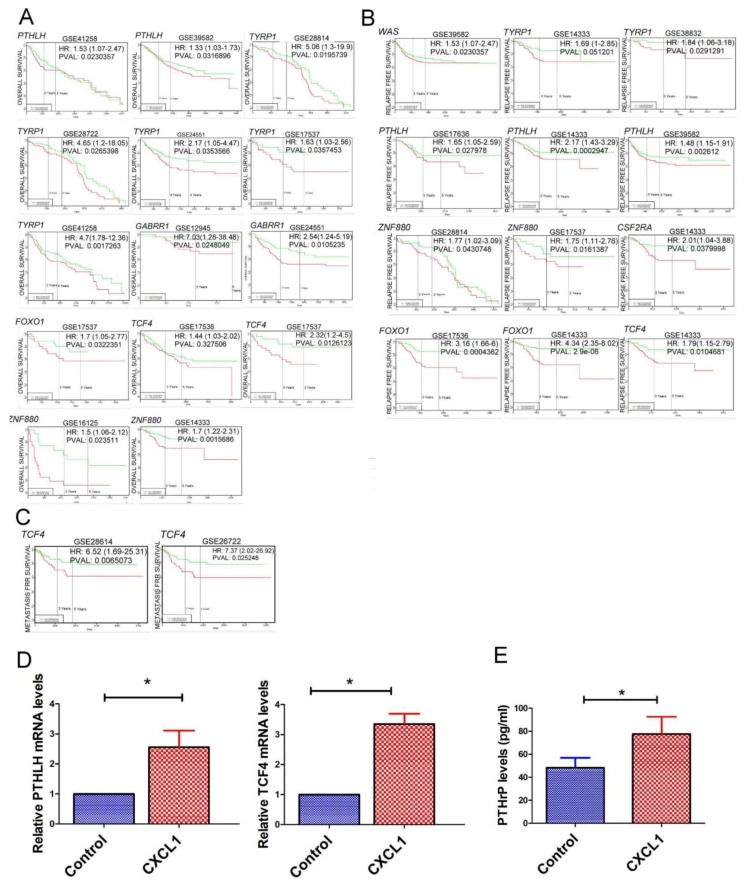
The correlation of CXCL1-regulated genes with CRC patient outcome. The correlation of overall survival (**A**), relapse-free (**B**) and metastasis-free rates (**C**) with CXCL-1 regulated genes. (**D**) The effect of CXCL1 in the expression of *PTHLH* and *TCF4* at mRNA levels. CXCL1 increased the expression of PTHrP (**E**) in SW620 cells. SW620 cells were treated with CXCL1 for 24 h, and the expressions of *PTHLH* and *TCF4* were determined by qRT-PCR and ELISA, respectively. Each value is the mean ± SD of three determinations. * Significant difference between the two test groups (*p* < 0.05) (*).

To investigate the molecular mechanism of TADC-derived CXCL1 in colon cancer progression, we assessed the transcriptome of CXCL1-treated SW620 cells by next generation sequencing (NGS). Significantly downregulated or upregulated genes were identified using a cut-off of *p* < 0.01 in combination with a fold-change >2. Compared to the control, 23 genes were upregulated and 21 genes were downregulated in CXCL1-treated SW620 cells (Table 1). We assessed upregulated genes obtained from RNA-seq data with regard to relevance to metastasis, relapse-free and overall survival rates of CRC patients via the bioinformatics website PROGgeneV2. High expression of *PTHLH*, *TYRP1*, *GABRR1*, *FOXO1*, *TCF4* and *ZNF880* correlated with reduced overall survival rates, while enhanced levels of *WAS*, *PTHLH*, *TYRP1*, *ZNF880*, *CSF2RA*, *FOXO1* and *TCF4* were associated with decreased relapse-free rates (Figure 5A,B). Increased expression of *TCF4* was correlated with a lower metastasis-free rate (Figure 5C). RT-qPCR and protein analysis revealed that *TCF4* and *PTHLH* expressions were upregulated in CXCL1-treated SW620 cells, at both the mRNA and protein levels (Figure 5D,E).

### 2.6. The Expression Pattern of microRNAs (miRNAs) in CXCL1-Treated Colon Cancer Cells

**Figure 6:**
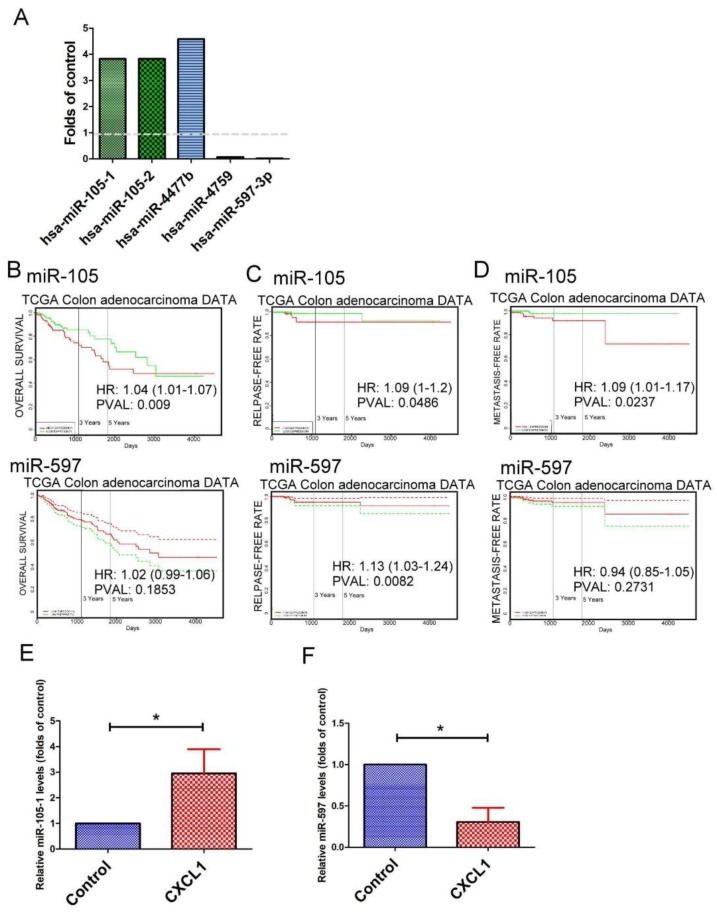
The effect of CXCL1 on the expression of microRNAs (miRNAs) in colon cancer. (**A**) The regulatory effect of CXCL1 on the expressions of miRNAs in colon cancer. Prognostic significance of miR-105 and miR-597 in overall survival (**B**), relapse-free (**C**) and metastasis-free rates (**D**) in colon cancer patients. The effect of CXCL1 on the levels of miR-105 (**E**) and miR-597 (**F**) in colon cancer. SW620 cells were treated with CXCL1 for 24 h, and the expression of miR-105 and miR-597 determined by qRT-PCR. Each value is the mean ± SD of three determinations. * Significant difference between the two test groups (*p* < 0.05) (*). Hsa, Homo sapiens; TCGA, The Cancer Genome Atlas; HR, hazard ratio; PVAL, *p*-value.

Concurrent with exon analysis, small fragment RNA samples from SW620 and CXCL1-treated SW620 cells were also processed for NGS. We found only five mature miRNAs (three miRNAs are up and two miRNAs are down) to be differentially expressed in CXCL1-treated SW620 cells when compared with the control (*p* < 0.001, fold-change > 2) (Figure 6A). We assessed the association between CXCL1-induced miRNA expression and patient outcome in CRC patients via the online database PROGmiRV2. Among these miRNAs, CRC patients with higher expression levels of miR-105-1 had significantly lower overall survival and metastatic-free rates in The Cancer Genome Atlas (TCGA) database (*p* < 0.05) (Figure 6B,C). However, no significant difference was found in the relapse-free survival of CRC patients with high or low expression of miR-105-1 (Figure 6D). RT-qPCR analysis reveals that miR-105 expression and miR-597-3p transcripts were upregulated and downregulated in CXCL1-treated SW620 cells (Figure 6E,F).

## 3. Discussion

**Figure 7:**
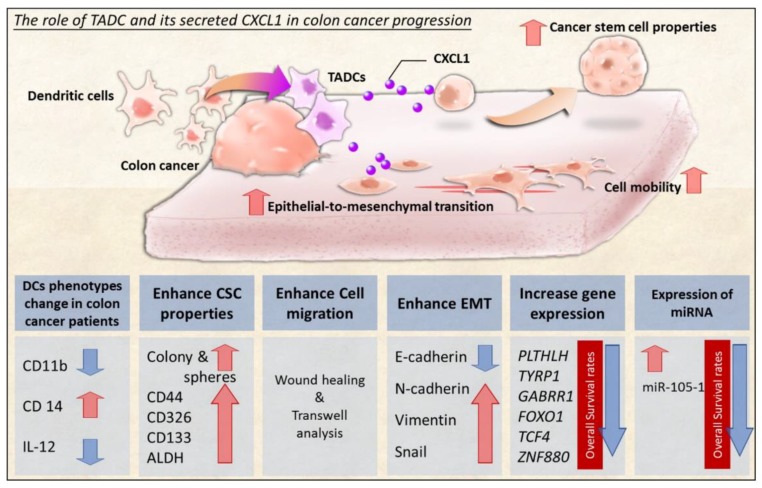
Proposed model of TADCs in colon cancer. Soluble factors in colon cancer cause abnormal phenotypes of dendritic cells, which in turn increase cancer stem cell properties, cell mobility, and EMT of colon cancer by producing the inflammatory chemokine CXCL1. Transcriptome analysis indicates that CXCL1 increases potential oncogene expression in colon cancer, including *PTHLH*, *TYRP1*, *FOXO1*, *TCF4*, *ZNF880* and the onco-miR miR-105. Our study indicates a new mechanism by which the colon cancer milieu exploits DC plasticity to support cancer progression.

The tumor microenvironment is widely known to be a vital factor in modulating cancer development [21,22]. Cells surrounding tumors consistently release various soluble factors, such as growth and inflammatory factors, which facilitate tumor progression [23,24]. In this study, we have demonstrated the contribution of colon cancer DC interaction to colon cancer progression. We demonstrated that colon cancer cells are able to change DC phenotype and anti-cancer cytokine IL-12 production. TADCs increased colon cancer CSC properties, cell migration and EMT by producing CXCL1 in a paracrine fashion (Figure 7). Investigation of the paradoxical roles of DCs in colon cancer development and the malignancy of tumors is a very important insight into the tumor microenvironment.

CXCL1 is a potent proinflammatory mediator of inflammatory diseases and infection, and is widely considered to both promote and exacerbate tumor growth and progression in several cancers [25,26]. CXCL1 is upregulated in various cancers and associated with cancer progression, such as cancer cell growth, proliferation, tumor angiogenesis and metastasis, after the activation of CXCR2 [27,28,29]. In addition, CXCL1 is not only involved in cancer progression, but also responsible for resistance to several chemotherapeutic drugs, such as oxaliplatin, doxorubicin, and cyclophosphamide [30,31,32,33]. CSCs are considered to display the clonogenic core of the cancer, since it is implied that these cells are involved in tumor propagation, progression, chemo-resistance and metastatic dissemination. In this study, we found that CXCL1 is not only released from malignant cells, but also secreted from cancer-conditioned DCs. TADCs express high levels of CXCL1, which in turn increases the tumorigenesis and chemo-resistance potential by increasing CSC-like properties. Furthermore, TADC-derived CXCL1 also enhances cancer migration and switches the epithelial phenotype to a mesenchymal characteristic, a key process of cancer metastasis. These findings suggest that TADC-derived CXCL1 may be a new candidate in conferring the ability for colon cancer to progress.

It is of interest to note that CXCL1-producing CD11c^+^ DCs were found to infiltrate the cancerous tissue of CT26-bearing mice. In addition, CD11c^+^ DCs isolated from patients with CRC produce high levels of CXCL1 when compared with CD11c^+^ DCs isolated from healthy donors. These results based on experimental cell studies, animal models, and clinical patients strongly suggest that TADCs are one of the critical effectors in CRC stroma enhancing the development of colon cancer by CXCL1 production.

PTHrP (gene symbol, *PTHLH*), an endocrine, paracrine and autocrine ligand, regulates cell differentiation and proliferation and is expressed in many tissues during their embryonic development, such as tooth, bone, and mammary gland maturation [34,35]. Cancer cells are one source of PTHrP, which causes humoral hypercalcemia and promotes the osteolytic bone metastases of a malignancy by activating the type 1 PTH/PTHrP receptor (PTH1R) in the kidneys and skeleton [36]. PTHrP is also implicated in the regulation of the behavior of primary cancer by the paracrine/autocrine action model, including cell proliferation, migration, and invasion, and prevents cell death in different types of cancers [37,38,39]. PTHrP supports the metastatic potential of prostate cancer by protecting tumor cells from anoikis in both in vitro and in vivo models [40]. PTHrP has also been associated with contributing to the lung colonization of colon cancer by inducing cell death in the endothelial cells of the lung microvasculature [41]. The current study demonstrated that in colon cancer, TADC-derived CXCL1 promotes expression of high levels of the *PTHLH* transcript and its protein, which are negatively correlated with relapse-free and overall survival rates in colon cancer. Our study provides an explanation as to why *PTHLH* is upregulated in colon cancer; however, further studies are required to extend our findings to other models of cancers.

Recently, miRNAs have attracted major interest, since they contribute to the precise regulation of various biological and pathological processes. Dysregulation of miRNAs has been observed in various stages of cancers, including tumorigenesis, progression, metastasis and tumor microenvironment remodeling [42,43]. Elevated miR-105 has been found in triple negative breast cancer, and positively correlated with poor survival rates [44]. Overexpression of miR-105 increases cell migration, invasion, stemness and resistance to cisplatin and chemoradiotherapy by downregulation of SFPR1. Cancer cells secrete exosomal miR-105, which is delivered to endothelial cells and destroys tight junctions and the integrity of blood vessels, resulting in distant cancer metastasis [45]. TNF-α, a well-known inflammatory cytokine contributing to cancer progression, dramatically stimulates miR-105 expression, inducing EMT in colon cancer [46]. Our results show that CXCL1 increases the expression of miR-105 expression, as determined both by small RNA-seq and qRT-PCR analysis. In addition, higher miR-105 expression indicates poorer metastasis-free rates and overall survival rates for colon cancer patients. These observations suggest that TADC-derived CXCL1 may increase stemness and cancer progression by increasing miR-105 in colon cancer via a paracrine loop. This, however, requires future research.

In conclusion, our results further support the role of TADCs and their secreted CXCL1 in colon cancer progression. The findings reported here not only introduce a novel mechanism of immunosuppression, but also provide preliminary evidence of the potential utility of CXCL1 inhibition as a therapeutic strategy in fighting cancer. Given that complement inhibition overrides tumor-dependent immunosuppression, this therapeutic approach may also hold promise as a supplement to anti-tumor vaccines.

## 4. Materials and Methods

### 4.1. Cell Cultures

The human colon cancer cell line SW620 and mouse colon cancer cell line CT26 were obtained from the American Type Culture Collection (ATCC) (Rockville, MD, USA). SW620 and CT26 cells were cultured in Leibovitz’s L-15 and RPMI-1640 medium, respectively. Both media were supplemented with 10% FBS and penicillin/streptomycin (100 U/0.1 mg/mL). All materials for cell culture were obtained from Thermo Fisher Scientific Inc. (Massachusetts, MA, USA). The cell lines used in the study were authenticated using short tandem repeat analysis with the Geneprint 10 System Kit (B9510, Promega, Madison, WI, USA) to confirm the identity, and tested for mycoplasma contamination every three months using mycoplasma test kits (Mycoalert Mycoplasma Detection Kit (Lonza Biologics, Portsmouth, NH, USA). Recombinant human CXCL1 protein was obtained from R&D systems (Minneapolis, MN, USA). The purity of recombinant human CXCL1 protein was more than 97% and endotoxin level is less than 0.01 EU/1 μg protein.

### 4.2. Flow Cytometry

The surface markers of DCs were analyzed using flow cytometry. The primary antibody against a specific surface marker or its corresponding isotype control was prepared in PBS containing 2% (*wt*/*vol*) bovine serum albumin for cell staining. The following antibodies were used: phycoerythrin (PE)-labeled anti-CD80 (Catalog #557227), CD1a (Catalog #555807), CD11c (Catalog #555392) and HLA-ABC (Catalog #555553) antibodies, fluorescein isothiocyanate (FITC)-labeled anti-HLA-DR (Catalog #555811) or anti-CD14 (Catalog #555397) antibodies, allophycocyanin (APC)-labeled anti-CD83 (Catalog #551073), and CD133 antibody (Catalog # 130-098-829), Brilliant™ Blue 515-labeled anti-CD11b (Catalog #564454), and Cy™5.5 labeled anti-CD86 (Catalog #561129) and CD209 (Catalog #558263) antibodies. All primary and isotype antibodies were purchased from BD Biosciences except CD133 antibody (MACS MicroBeads; Miltenyi Biotec Ltd., Bergisch Gladbach, Germany). Flow cytometric data were acquired by an Accuri C6 flow cytometer and analyzed using CellQuest software (BD Biosciences, San Jose, CA, USA).

ALDH activity was assessed using an ALDEFLUOR kit (Stem Cell Technologies, Vancouver, BC, Canada). Briefly, cells were incubated in Aldefluor assay buffer containing ALDH substrate (1 μmol/L per 1 × 10^6^ cells), with or without ALDH inhibitor diethylaminobenzaldehyde (DEAB) at 37 °C for 30 min; ALDH activity was then measured using a flow cytometer.

### 4.3. Quantitative Reverse Transcriptase-Polymerase Chain Reaction (qRT-PCR)

Total RNAs were extracted with TRIzol reagent (Thermo Fisher Scientific) and reverse transcribed into cDNA using an oligo (dT) primer and reverse transcriptase (Takara, Shiga, Japan) following the manufacturer’s protocols. The reverse transcription of miRNAs into cDNA was performed using the Mir-X™ miRNA First Strand Synthesis Kit (Clontec). The expression levels of specific genes were determined by a StepOne-Plus machine (Applied Biosystems, Foster City, CA, USA), using real-time analysis with SYBR Green (Thermo Fisher Scientific, Waltham, MA, USA). The following primers were used: PTHLH (forward, 5′-GGTGTTCCTGCTGAGCTACG-3′ and reverse, 5′-TCTGCGATCAGATGGTGAAG-3′); CXCL1 (forward, 5′-AGGGAATTCACCCCAAGAAC-3′ and reverse, 5′-TAACTATGGGGGATGCAGGA-3′); CXCL2 (forward, 5′-GCAGGGAATTCACCTCAAGA-3′ and reverse, 5′-AGCTTCCTCCTTCCTTCTGG-3′); CXCL3 (forward, 5′-GCAGGGAATTCACCTCAAGA-3′ and reverse, 5′-TCTGAACCATGGGGGATG-3′); miRNA-105 (TCAAATGCTCAGACTCCTGTGGT); GAPDH (glyceraldehyde 3-phosphate dehydrogenase) (forward, 5′-TTCACCACCATGGAGAAGGC-3′ and reverse, 5′-GGCATGGACTGTGGTCATGA-3′) and U6 (forward, TGGAACGCTTCACGAATTTGCG; reverse, GGAACGATACAGAGAAGATTAGC). Relative expression levels of the cellular mRNA and miRNA were normalized to GAPDH or U6 snRNA, respectively. Quantitative analysis was normalized to GAPDH, or U6 was performed in accord with the comparative cycle threshold (Ct) method.

### 4.4. Differentiation of DCs

Peripheral blood mononuclear cells (PBMCs) of healthy donors were isolated using Ficoll-Hypaque gradient (GE Healthcare Bio-Sciences, Little Chalfont, UK), and CD14^+^ monoclonal antibody-conjugated magnetic beads (MACS MicroBeads; Miltenyi Biotec Ltd, Bergisch Gladbach, Germany) were used for CD14^+^ monocyte isolation from PBMCs, following the manufacturer’s instructions. CD14^+^ monocytes were cultured in RPMI-1640 medium containing 50% L-15 (for control) or SW620-CM (for TADCs), in the presence of 10 ng/mL GM-CSF and IL-4 (R&D Systems, Minneapolis, MN, USA) for five days to generate monocyte-derived DCs or TADCs. The study protocol was approved by the Institutional Review Board (IRB) of Kaohsiung Medical University Hospital (Kaohsiung, Taiwan), and all participants provided written informed consent in accordance with the Declaration of Helsinki before entering the study.

### 4.5. Animal Tumor Model

Six-week-old male inbred mice of BALB/cByJNarl strain were purchased from the National Laboratory Animal Center (Taiwan) and maintained in pathogen-free conditions. Mouse colon cancer CT26 cells (1 × 10^6^ cells) were injected into mice (*n* = 6 for each group) via intraperitoneal injection. Fourteen days after tumor cell injection, the peritoneal lymph nodes were harvested for CD11c+ cell isolation using CD11c monoclonal antibody-conjugated magnetic beads (MACS MicroBeads; Miltenyi Biotec Ltd, Bergisch Gladbach, Germany). The animal study was approved by the Animal Care and Use Committee at Kaohsiung Medical University (IACUC Approval No: 102110, 18 December 2013 approved).

### 4.6. Measurement of Secreted Factors

Supernatants from the CD11c^+^ cells, DCs or TADCs were collected, and CXCL1, IL-10 and IL-12 levels were determined by Milliplex MAP kit (Millipore, Billerica, MA, USA). The expressions of various MMP in SW620 cells were determined by Luminex performance human MMP kit (R&D Systems, Minneapolis, MN, USA). Mouse CXCL1 and human PTHLH levels were assessed and quantified using mouse CXCL1 (R&D Systems) and human PTHLH ELISA kit (Casabio), respectively.

### 4.7. Soft Agar Colony Formation, Tumor Spheroid and Cell Migration Analysis

Equal numbers of cells were seeded onto an 0.6% agarose base in a 0.3% top soft agar layer in a 24-well plate. Cells were treated with or without CXCL1 (10 ng/mL) and incubated for 40 days, and the total number of colonies in each well was counted. Colonies were quantified microscopically under 10× magnification. SW620 cells were seeded in a 24 well plate until confluence and the wounds in cell monolayers were created by scraping with a sterile micropipette tip. Cells were treated with CXCL1 (10 ng/mL) during the wound healing assay and the wound closure was observed after 72 h. A quantitative migration assay was performed using the transwell system QCM 24-well (Millipore Corp., Billerica, MA, USA). Briefly, SW620 cells (1 × 10^5^/well) were seeded into the top chamber, and CXCL1 added to the bottom wells as chemoattractant for 24 h. At the end of the treatment, cells were post-stained with crystal violet. SW620 cells were seeded in ultra-low attachment plates (Corning Life Sciences, NY, USA), with or without CXCL1 for tumor sphere formation. After 10 days, the tumor spheres were assessed by microscopy or dissociated into single cells for surface marker or ALDEFLUOR analysis.

### 4.8. Immunoblot Analysis

Total proteins of SW620 cells were extracted using RIPA lysis buffer (Millipore, Billerica, MA, USA). SDS-PAGE electrophoresis was performed with equivalent amounts of protein lysates, followed by protein transfer to polyvinylidene fluoride (PVDF) membranes. The membranes were blocked in Tris-buffered saline containing 0.05% Tween 20 (TBST) and 5% non-fat powdered milk, and incubated with primary antibodies at 4 °C overnight. After washing with TBST three times, the membranes were then incubated with horseradish peroxidase-labeled secondary antibody for 1 h. After corresponding secondary antibody incubation, the immunoreactivity was detected using an enhanced chemiluminescence substrate (Millipore) on an imaging capture system (Alpha Innovation). Antibodies against OCT4 (Catalog # 2890), Nanog (Catalog #4903), c-Myc (Catalog # 5605), SOX2 (Catalog # 3579) and GAPDH (Catalog #5174) were obtained from Cell Signaling Technology (Beverly, MA, USA). Anti-N-cadherin (Catalog # 610921), E-cadherin (Catalog # 610182) and vimentin (Catalog # 550513) antibodies were obtained from BD Biosciences (San Jose, CA, USA). The quantitation result of the immunoblot was performed by AlphaImager software (Alpha Innotech, San Leandro, CA, USA).

### 4.9. Next Generation Sequencing and Bioinformatics Analysis

The deep sequencing of RNA and small RNA was carried out at a biotechnology company (Welgene, Taipei, Taiwan) using the Solexa platform. RNA and small RNA library construction was carried out using an Illumina sample preparation kit, following the protocol of the TruSeq RNA or Small RNA Sample Preparation Guide. The correlation between gene expression and clinical outcome of colon cancer patients was evaluated by PROGgeneV2 (http://watson.compbio.iupui.edu/chirayu/proggene/database/index.php) (access on 11 April 2018) and PROGmiRV2 (http://xvm145.jefferson.edu/progmir) (access on 8 May 2018), which are tools for identifying prognostic RNAs and miRNA biomarkers in multiple cancers using publicly available data [47,48].

### 4.10. Statistical Analysis

Data were expressed as mean ± SD. Unpaired Student’s *t*-test was used to compare the difference between the control and experimental groups, and one-way ANOVA with Tukey’s post hoc analysis was used for multiple comparisons among groups. A *p*-value < 0.05 was considered to be a statistically significant difference between groups. All statistical analysis was carried out using the program GraphPad Prism version 5.03 (GraphPad Software, San Diego, CA, USA).

## Figures and Tables

**Table 1:** Gene Profile of CXCL1-Treated SW620 Cells.

Gene Name	Ratio (CXCL/Control)	Log2 Ratio	*p* Value
*WAS*	2.75575	1.46245	0.28025
*PTHLH*	3.09636	1.63057	0.00285
*TYRP1*	3.48983	1.80316	0.0775
*TRIM16L*	2.21167	1.14514	0.09035
*TFEB*	2.5323	1.34045	0.07635
*CSTA*	3.14503	1.65307	0.1503
*AOAH*	2.95457	1.56295	0.0516
*GABRR1*	2.62297	1.3912	0.03455
*FOXO1*	2.7327	1.45032	0.10195
*SPAG17*	2.55284	1.35211	0.31355
*SCNN1D*	2.31426	1.21055	0.29645
*DYNLRB2*	2.77593	1.47297	0.1716
*RP11-231C14.4*	3.51186	1.81223	0.00315
*WFDC9*	2.10763	1.07562	0.2166
*TCF4*	2.29962	1.20139	0.05605
*CSF2RA*	2.05608	1.03989	0.1571
*ZNF880*	2.10466	1.07359	0.18095
*H2BFS*	3.42202	1.77485	0.13455
*LY6G6D*	2.55342	1.35243	0.5991
*CTC-435M10.3*	2.42044	1.27527	0.36275
*BCL2L2-PABPN1*	4.05028	2.01802	0.3231
*RP11-438J1.1*	2.75195	1.46046	0.21695
*CH17-270A2.2*	3.22723	1.6903	0.6751
*ABCB4*	0.34488	−1.5358	0.05905
*NME1-NME2*	0.28497	−1.8111	0.5181
*MAN2B2*	0.484	−1.0469	0.18655
*POU2F2*	0.19867	−2.3315	0.1448
*DGKG*	0.34246	−1.546	0.12485
*HPX*	0.07198	−3.7963	0.1496
*GBP3*	0.21884	−2.1921	0.00015
*GRPR*	0.45999	−1.1203	0.01765
*KHDRBS3*	0.25515	−1.9706	0.0735
*ZCCHC2*	0.47568	−1.0719	0.0612
*CYP17A1*	0.41877	−1.2558	0.1948
*ALS2CR12*	0.3871	−1.3692	0.05095

## Abbreviations

CM	Condition medium
CRC	Colorectal cancer
CSC	Cancer stem cell
CXCL1	chemokine (C-X-C motif) ligand 1
CXCR2	chemokine (C-X-C motif) receptor 2
DCs	Dendritic cells
EMT	Epithelial-to-mesenchymal transition
NGS	next generation sequencing
PBMCs	Peripheral blood mononuclear cells
TADCs	Tumor-associated dendritic cells
